# PubMed's core clinical journals filter: redesigned for contemporary clinical impact and utility

**DOI:** 10.5195/jmla.2023.1631

**Published:** 2023-07-10

**Authors:** Michele Klein-Fedyshin, Andrea M. Ketchum

**Affiliations:** 1 kleinf@pitt.edu, Research & Clinical Instruction Librarian, Health Sciences Library System, University of Pittsburgh, Pittsburgh, PA.; 2 ketchum@pitt.edu, Emeritus, Health Sciences Library System, University of Pittsburgh, Pittsburgh, PA.

**Keywords:** MEDLINE, clinical medicine, databases, bibliographic, evidence-based medicine, periodicals as topic, PubMed

## Abstract

**Objective:**

The Core Clinical Journals (CCJ) list, produced by the U.S. National Library of Medicine (NLM), has been used by clinicians and librarians for half a century for two main purposes: narrowing a literature search to clinically useful journals and identifying high priority titles for library collections. After documentation of low usage of the existing CCJ, a review was undertaken to assess current validity, followed by an update to current clinical needs.

**Methods:**

As the subject coverage of the 50-year-old list had never been evaluated, the CCJ committee began its innovative step-wise approach by analyzing the existing subject scope. To determine whether clinical subjects had changed over the last half-century, the committee collected data on journal usage in hospitals and medical facilities, adding journal usage from Morning Report blogs recording the journal article citations used by physicians and residents in response to clinical questions. Patient-driven high-frequency diagnoses and subjects added contextual data by depicting the clinical environment.

**Results:**

The analysis identified a total of 80 subjects and selected 241 journals for the updated Clinical Journals filter, based on actual clinical utility of each journal.

**Discussion:**

These data-driven methods created a different framework for evaluating the structure and content of this filter. It is the real-world evidence needed to highlight CCJ clinical impact and push clinically useful journals to first page results. Since the new process resulted in a new product, the name warrants a change from Core Clinical Journals (CCJ) to Clinically Useful Journals (CUJ). Therefore, the redesigned NLM Core Clinical Journals/AIM set from this point forward will be referred to as Clinically Useful Journals (CUJ). The evidence-based process used to reframe evaluation of the clinical impact and utility of biomedical journals is documented in this article.

## INTRODUCTION

For fifty years, from 1970-2020, clinicians and librarians used either the Abridged Index Medicus (AIM) or Core Clinical Journals (CCJ) list for two main purposes: narrowing a literature search to clinically important journals and identifying high-priority titles for medical library collections. Originally developed as a manageable subset of 100 important titles from the 2,300 English-language journals then indexed in Index Medicus, now known as MEDLINE (both produced by the U.S. National Library of Medicine (NLM), the Abridged Index Medicus (AIM) list aided clinicians seeking to limit their searches to clinically oriented articles. Investigation revealed that there was no record of the methods used to produce the 1970 AIM list other than published reports of the professions involved (librarians, physicians, editors) and that it was designed for practicing physicians [1]. In 1979, 26 journals were added (along with one in 1978), and eight titles were deleted, resulting in 119 indexed journals [2]. NLM automated and renamed it the Core Clinical Journals filter to augment PubMed.gov in 2001 [3]. Subsequently, one title was removed to leave 118 journals on the CCJ list [4].

The impetus for the current update was the 2014 research and subsequent article demonstrating the CCJ filter's low usage, recall and precision for clinical searching [5], specifically the findings that only 30% of clinically used articles were from the CCJ filter and only 16% of the journals were represented in Core titles. In 2015, the Medical Library Association (MLA) convened an Ad Hoc Committee by request of the U.S. National Library of Medicine (NLM) and charged it to produce a new Core Clinical Journals list of immediate interest to healthcare practitioners and hospital librarians who require access to essential clinical literature (Appendix A).

The Ad Hoc committee was cochaired by Michele Klein-Fedyshin, MSLS, BSN, RN, and Andrea M. Ketchum, MS, MLIS, and included members from the Hospital Library and Nursing and Allied Health Sections of MLA, as well as other specialties, such as Medical Informatics. Only five members were journal “Selectors,” although the committee included liaisons from the MLA Board and NLM. The committee recognized that health care professionals need to conduct efficient, yet effective searches. By evaluating a broader variety of health care professions, hospital/outpatient/office environments, and patient ages and conditions, the new list would be valuable to all clinical practitioners.

## METHODOLOGY

Since the subject coverage of the 50-year-old list had never been evaluated, the committee formulated a data-driven, step-wise approach to the update, selecting subjects first and journals second. This approach generated two questions:

1. Do NLM subject headings represented by the existing CCJ list align with current clinical practice?
2. What journals currently indexed in MEDLINE best meet newly aligned CCJ subject headings-defined usage and practice criteria?

### Initial Data Gathering Steps of Subject and Journal Selection Process

Lacking any previous methodology for the process, the Committee considered data sources, scope, and usage statistics for both subject and journal selection criteria. A totally new, data-driven approach incorporating clinical Journal Usage (JU) and Patient-Driven Count (PDC) indicators was developed to demonstrate actual clinical activity and journal use.

Table 1 contains a summary of the steps taken. Additional details follow.

**Table 1:** Methodology for Evaluating Coverage of Clinical Literature

7-Step Methodology for Evaluating Coverage of Clinical Literature	
1.	Compile journal usage (JU) data collected from hospitals'/health systems' institutional libraries and Morning Report blogs
2.	Evaluate current trends, topics, diseases being treated, and goals by gathering data from government (e.g., Healthy People 2020, discharge diagnosis statistics), industry (e.g., Medscape), and trustworthy health sciences sources (e.g., Doody's Core Titles subjects).
3.	Using the Broad Subject Headings of MeSH, classify the journals used and the topics of data from #2 above to compile JU data and Patient Driven Counts (PDC) / Indicators.
4.	Using the JU's and PDCs, calculate the 25% and 75% thresholds for JU and PDC to divide them into High, Medium and Low categories of subjects potentially needing coverage.
5.	Eliminate those Broad Subjects with low JU's, preclinical, animal, or veterinary sciences.
6.	Calculate number of journals needed per Broad Subject using the proportion method and parallel analysis.
7.	In needed Broad Subjects, rank journals by frequency of usage (JU). Select the number of journals needed for that subject.

JUs represent the number of times journals were used in health facilities, rather than a tally of subscriptions. JU data was sourced from healthcare facility data. MLA institutional members were contacted via both email and the MLA newsletter for journal use statistics in clinical environments from 2009-2015. Responses with usage from the University of Pittsburgh Health Sciences Library System and Louisiana State University Health Sciences Center at Shreveport were augmented by the Kaiser Permanente health system data. The Kaiser Permanente Medical Groups and Kaiser Foundation Hospitals system includes 39 hospitals, 734 medical offices, over 23,000 physicians and 65,000 nurses over 8 states and the District of Columbia [6]. In addition, Morning Report Blog journal usage from the US and Canada (Appendix B), such as the UCSF Internal Medicine Morning Report Blog, was included in Journal Usage counts. A national survey of 32 Primary Access Libraries' (PALS) journal usage added to the background JU data. PALS Libraries are hospital libraries or other non-academic health sciences libraries belonging to the NLM's Regional Medical Library (RML) network, ensuring clinical relevance. Together they represent reports from over 814 clinical locations in the United States and Canada.

PDCs include national discharge statistics by diagnosis gathered by the Healthcare Cost and Utilization Project (HCUP) [7] from the U.S. Agency for Healthcare Research and Quality (AHRQ) along with contemporary clinical concerns identified through Doody Core Titles' subject headings (used with permission) [8], Healthy People 2020 Objectives [9] for national contemporary health concerns, and topic frequency data for requested alerts in Medscape [10] (Appendix C).

Thus, real-world evidence incorporating practical journal usage by a wide variety of institutions and professionals correlated with national U.S. clinical data and 2020 health goals ultimately produced two tools: JUs paired with PDCs to rank updated clinical subjects, and a “clinical utility score” to indicate a journal's current clinical usefulness. Together, they enabled the subject and journal selection process.

## SUBJECT REVIEW

### The Committee Evaluated CCJ Subject Coverage First

MEDLINE journals are indexed with a simplified list of 125 NLM Broad Subject Headings [11], which serve to aggregate several separate MeSH headings. One or more Broad Subject Headings are provided in the PubMed bibliographic record for every MEDLINE journal, and many are assigned multiple Broad Subject Headings. After the JU and PDC counts were gathered, the data were correlated by the statistician for all 125 Broad Subject Headings assigned across CCJ journals. The correlation of JU and PDC data divided those Subject Headings into nine paired high, middle and low count groups.

Table 2 illustrates how the correlation divided the Subject Headings and illustrates their application.

**Table 2:** Correlation of JU and PDC usage counts to rank Broad Subject Heading

Variables: 1. Journal Usage (JU) 2. Patient Driven Counts (PDC) Cut-off points: Quartiles 1 and 4	**Quartile, %**	**Journal Usage (JU)**	**Patient Driven Counts (PDC)**
	**Q1 0%**	**1**	**1**
	**Q2 25%**	**936.5**	**2**
	**Q3 75%**	**52,739**	**866,810.25**
	**Q4 100%**	**1,538,830**	**11,664,724**
	**High PDC 100%** **11,664,724** 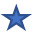	**Middle PDC 75%** **866,810** 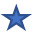	**Low PDC 25%** **2** 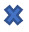
**High JU** 100% 1,538,830 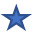	High – High 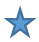	High – Middle 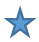	High – Low 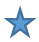
**Middle JU 75%** **52,739**	Middle – High 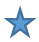	Middle – Middle 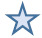	Middle – Low 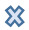
**Low JU 25%** **936** 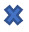	Low-High 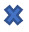	Low – Middle 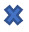	Low-Low 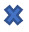

Key: Bold Star = Keep subject; Bold X = Reject subject; Unbolded = Discretionary; Unbolded = Discretionary

The resulting formal Subject Selection Criteria for CUJ coverage that the committee used are:

Keep all subjects with either high journal usage or high patient-driven counts unless JU<1000

1. Delete all subjects with JU<1000
2. Delete all subjects relating to animals
3. Delete any remaining preclinical sciences (e.g., Cell Biology)

In all, 80 subjects were incorporated into the new CUJ coverage and 45 were omitted, primarily due to the clinical inclusion criteria. Although most selected subjects previously had journals in CCJ, it was notable that 33 existing subjects previously had no journals assigned, among them contemporary healthcare topics such as Anti-Infective Agents, Medical Genetics, Nephrology, and Women's Health. Increased Mental Health and Substance Abuse coverage enhances the immediate relevance of the new CUJ filter. Details of the calculations are in Appendix D. The complete subject analysis of final CUJ subjects is reported in Appendix E.

## JOURNAL SELECTION FOR THE QUALIFYING SUBJECTS

### Deciding How Many Journals Were Needed per Subject

Determining the number of journals needed involved two methods per subject, the results of which were merged. The first calculation was the Proportion Method. It first derived the proportion of the original number of CCJ Journals to the number of MEDLINE journals indexed at that time. Reflecting overall literature growth, this percentage was applied to the current number of MEDLINE journals to find a target number of journals needed for the new CUJ list. To determine how many of this target number should be allocated to each subject, we calculated that subject's percentage of overall clinical use in our JU data. That percentage of clinical use per subject was multiplied by the target number of journals to derive a count of journals needed for each subject. Since this method used MEDLINE expansion as one component, a second method of Parallel Analysis was suggested to account for other factors.

Proportion Method: The first method calculated what percentage the prior Abridged Index Medicus constituted of primarily English language MEDLINE journals in the conception year of 1970. This calculation was 100 Core journals/2,300 primarily English language MEDLINE journals. This result (0.044) was then applied to the primarily English language, MEDLINE journals indexed in 2018, or 5152, for a total of 226. However, the committee decided on 222 journals designated for the new CUJ list to account for possible growth in nonclinical literature. To determine how many were allocated to each of 80 subjects, we determined each subject's percentage of clinical use and multiplied by 222. As an example, the subject Medicine's percentage of journal usage was 6.425% of the total. This percentage was multiplied by the 222 target to give 14 needed journals to cover this largest category.

A Parallel Analysis augmented the Proportion Method. Parallel Analysis is a statistical method to determine components to use in a principal component analysis (PCA) or factor analysis. We analyzed four components: Journal Usage, Subject Frequency, Patient-Driven Counts and Elsevier's Source Normalized Impact per Paper (SNIP) score [12], a citation metric standardized across subject fields to permit nonbiased comparison. (See Appendix D, Figures/Table 1-6 for added statistical details.) The two methods of calculating journals needed per subject were merged to recommend the maximum number of journals for each subject.

### Assigning Selectors and Creating Uniform Criteria

To facilitate the selection of journal titles for each subject, the committee co-chairs organized the subjects into logical groupings so that the same person covered similar subjects (e.g., psychiatry and psychology).

### Candidate Journal Selection

Two versions of a Candidate Journal Worksheet applicable across all subjects were created: a working version to use for collecting comparative data for each subject, and a final version with just the selected journal titles. Candidate journal data were recorded on the subject worksheet, and journals in each subject were ranked. Figure 2 depicts a Candidate Journal Worksheet.

**Table 3:** Candidate Journal Worksheet

**Subject** **Selector:**	**Max# Journals:** **Rank:**	**Raw Count** **Clinical Use**	**% Clinical Use**	**NLM ID**
**Journal**				
**Journal**				
**Journal**				

The following “ground rules” for candidate journals were set:

1. The journal title must be currently indexed in MEDLINE;
2. Numeric ranking among our clinically used titles derived from JU counts was critical;
3. Must be indexed to the Broad Subject Heading under consideration;
4. The maximum number of journals allocated for that subject could not be exceeded; fewer recommended journals were allowable if usage data did not support reaching that number.

A detailed description of the calculations used to determine subject coverage and select journals for the resulting subjects is in Appendix D. A decision toolkit consisting of Candidate Journal Worksheets, Journal Usage counts, and PALS ranking helped all selectors use common factors when picking journal titles for each assigned subject. Of note, only Ad Hoc CCJ committee members who were not NLM or MLA staff could select journals.

## RESULTS

### Final Clinically Useful Journals List and Recommendations

**Figure 1:**
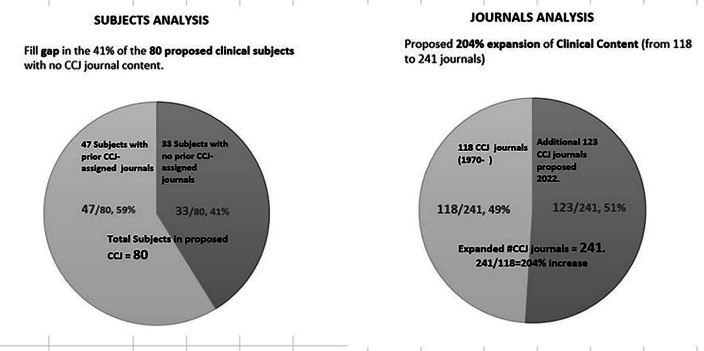
Subjects, Journals Analyses

The new Clinically Useful Journals list adds journals for the 33 new subjects. Coverage expanded in some of the 47 existing subjects. Figure 1 graphically depicts these changes.

The new analysis and selection process resulted in a 241-journal product named Clinically Useful Journals (CUJ). The full alphabetical list of journals comprising the new CUJ is displayed below in Table 4.

**Table 4:** Proposed CUJ 241 Journal Titles in Alphabetical Order

1	*AACN Advanced Critical Care*
2	*Academic Emergency Medicine*
3	*Academic Medicine*
4	*Addictive Behaviors*
5	*Age and Ageing*
6	*AJR. American Journal of Roentgenology*
7	*Allergy*
8	*American Family Physician*
9	*American Heart Journal*
10	*American Journal of Epidemiology*
11	*American Journal of Gastroenterology*
12	*American Journal of Hematology*
13	*American Journal of Kidney Diseases*
14	*American Journal of Medical Genetics. Part A*
15	*American Journal of Medicine*
16	*American Journal of Nursing*
17	*American Journal of Obstetrics and Gynecology*
18	*American Journal of Preventive Medicine*
19	*American Journal of Psychiatry*
20	*American Journal of Respiratory and Critical Care Medicine*
21	*American Journal of Sports Medicine*
22	*American Journal of Surgical Pathology*
23	*American Journal of the Medical Sciences*
24	*Anesthesia and Analgesia*
25	*Annals of Allergy, Asthma, and Immunology*
26	*Annals of Emergency Medicine*
27	*Annals of Internal Medicine*
28	*Annals of Neurology*
29	*Annals of Oncology*
30	*Annals of Pharmacotherapy*
31	*Annals of Surgery*
32	*Annals of Surgical Oncology*
33	*Annals of the Rheumatic Diseases*
34	*Annals of Thoracic Surgery*
35	*Archives of Disease in Childhood*
36	*Archives of Disease in Childhood. Fetal and Neonatal edition*
37	*Archives of Physical Medicine and Rehabilitation*
38	*Arthritis & Rheumatology*
39	*Arthritis Care & Research*
40	*Arthroscopy: Journal of Arthroscopic and Related Surgery*
41	*Autoimmunity Reviews*
42	*Best Practice and Research. Clinical Rheumatology*
43	*Biological Psychiatry*
44	*BJOG: an international journal of obstetrics and gynaecology*
45	*BJU International*
46	*Blood*
47	*BMJ (Clinical research ed.)*
48	*Bone Marrow Transplantation*
49	*Brain: a journal of neurology*
50	*Breastfeeding Medicine*
51	*British Journal of Anaesthesia*
52	*British Journal of Cancer*
53	*British Journal of Dermatology*
54	*British Journal of Haematology*
55	*British Journal of Ophthalmology*
56	*British Medical Bulletin*
57	*CA: a Cancer Journal for Clinicians*
58	*Cancer*
59	*Cancer Treatment Reviews*
60	*Catheterization and Cardiovascular Interventions*
61	*Chest*
62	*Circulation*
63	*Clinical Biochemistry*
64	*Clinical Biomechanics*
65	*Clinical Gastroenterology and Hepatology*
66	*Clinical Infectious Diseases*
67	*Clinical Obstetrics and Gynecology*
68	*Clinical Pharmacology and Therapeutics*
69	*Clinical Therapeutics*
70	*Clinics in Podiatric Medicine and Surgery*
71	*CMAJ: Canadian Medical Association Journal*
72	*Computers, Informatics, Nursing: CIN*
73	*Critical Care Medicine*
74	*Current Opinion in Cardiology*
75	*Current Opinion in Gastroenterology*
76	*Current Opinion in Nephrology and Hypertension*
77	*Current Opinion in Pediatrics*
78	*Current Opinion in Rheumatology*
79	*Diabetes Care*
80	*Diabetes Research and Clinical Practice*
81	*Diagnostic Microbiology and Infectious Disease*
82	*Digestive Diseases and Sciences*
83	*Diseases of the Colon and Rectum*
84	*Drug and Alcohol Dependence*
85	*Early Human Development*
86	*Epilepsia*
87	*Epilepsy and Behavior*
88	*Europace*
89	*European Heart Journal*
90	*European Journal of Cancer*
91	*European Journal of Cardiothoracic Surgery*
92	*European Journal of Heart Failure*
93	*European Journal of Internal Medicine*
94	*European Journal of Nuclear Medicine and Molecular Imaging*
95	*European Journal of Radiology*
96	*European Urology*
97	*Fertility and Sterility*
98	*Gastroenterology*
99	*Gastrointestinal Endoscopy*
100	*Gut*
101	*Gynecologic Oncology*
102	*Head & Neck*
103	*Headache: The Journal of Head and Face Pain*
104	*Health Affairs*
105	*Heart (British Cardiac Society)*
106	*Heart Rhythm*
107	*Hepatology*
108	*Human Pathology*
109	*Human Reproduction*
110	*Hypertension*
111	*Infection Control and Hospital Epidemiology*
112	*International Journal of Antimicrobial Agents*
113	*International Journal of Cancer*
114	*International Journal of Cardiology*
115	*International Journal of Clinical Practice*
116	*International Journal of Obesity*
117	*International Journal of Radiation Oncology, Biology, Physics*
118	*International Urogynecology Journal*
119	*JAMA*
120	*JAMA Dermatology*
121	*JAMA Internal Medicine*
122	*JAMA Neurology*
123	*JAMA Ophthalmology*
124	*JAMA Otolaryngology–Head & Neck Surgery*
125	*JAMA Pediatrics*
126	*JAMA Psychiatry*
127	*JAMA Surgery*
128	*Journal for Healthcare Quality*
129	*Journal of Acquired Immune Deficiency Syndromes: JAIDS*
130	*Journal of Advanced Nursing*
131	*Journal of Allergy and Clinical Immunology*
132	*Journal of Alternative and Complimentary Medicine*
133	*Journal of Bone and Joint Surgery. American Volume*
134	*Journal of Cardiac Failure*
135	*Journal of Clinical Endocrinology and Metabolism*
136	*Journal of Clinical Gastroenterology*
137	*Journal of Clinical Neuroscience*
138	*Journal of Clinical Oncology*
139	*Journal of Clinical Pathology*
140	*Journal of Clinical Psychology*
141	*Journal of Clinical Psychopharmacology*
142	*Journal of Emergency Medicine*
143	*Journal of Foot and Ankle Surgery*
144	*Journal of General Internal Medicine*
145	*Journal of Hand Surgery*
146	*Journal of Hepatology*
147	*Journal of Hospital Infection*
148	*Journal of Hospital Medicine*
149	*Journal of Infection*
150	*Journal of Infectious Diseases*
151	*Journal of Internal Medicine*
152	*Journal of Investigative Dermatology*
153	*Journal of Medical Genetics*
154	*Journal of Midwifery and Women's Health*
155	*Journal of Neurology, Neurosurgery, and Psychiatry*
156	*Journal of Nursing Administration*
157	*Journal of Obstetric, Gynecologic, and Neonatal Nursing: JOGNN*
158	*Journal of Occupational and Environmental Medicine*
159	*Journal of Oral and Maxillofacial Surgery*
160	*Journal of Orthopaedic and Sports Physical Therapy*
161	*Journal of Orthopaedic Trauma*
162	*Journal of Pain and Symptom Management*
163	*Journal of Palliative Medicine*
164	*Journal of Pediatric Gastroenterology and Nutrition*
165	*Journal of Pediatric Hematology/Oncology*
166	*Journal of Pediatric Orthopedics*
167	*Journal of Pediatric Surgery*
168	*Journal of Perinatology*
169	*Journal of Psychopharmacology*
170	*Journal of Substance Abuse Treatment*
171	*Journal of Surgical Oncology*
172	*Journal of the American College of Cardiology: JACC*
173	*Journal of the American Geriatrics Society*
174	*Journal of the American Medical Directors Association*
175	*Journal of the American Medical Informatics Association: JAMIA*
176	*Journal of the National Cancer Institute*
177	*Journal of Thoracic and Cardiovascular Surgery*
178	*Journal of Thrombosis and Haemostasis*
179	*Journal of Trauma and Acute Care Surgery*
180	*Journal of Urology*
181	*Journal of Vascular Surgery*
182	*JPEN. Journal of Parenteral and Enteral Nutrition*
183	*Kidney International*
184	*Lancet*
185	*Laryngoscope*
186	*Leukemia*
187	*Liver Transplantation*
188	*Medical Care*
189	*Medical Clinics of North America*
190	*Medical Letter on Drugs and Therapeutics*
191	*Medicine (Baltimore)*
192	*Modern Pathology*
193	*Molecular Genetics and Metabolism*
194	*Movement Disorders*
195	*Muscle and Nerve*
196	*Nephrology, Dialysis, Transplantation*
197	*Neurology*
198	*Neurosurgery*
199	*New England Journal of Medicine*
200	*Nursing*
201	*Obesity*
202	*Obstetrical and Gynecological Survey*
203	*Obstetrics and Gynecology*
204	*Oral Surgery, Oral Medicine, Oral Pathology and Oral Radiology*
205	*Otolaryngology-Head and Neck Surgery*
206	*Pain*
207	*Pain Medicine*
208	*Patient Education and Counseling*
209	*Pediatric Dermatology*
210	*Pediatric Infectious Disease Journal*
211	*Pediatrics*
212	*Pharmacoepidemiology and Drug Safety*
213	*Plastic and Reconstructive Surgery*
214	*Postgraduate Medical Journal*
215	*Preventive Medicine*
216	*Primary Care: Clinics in Office Practice*
217	*Psychiatric Services*
218	*QJM: monthly journal of the Association of Physicians*
219	*Radiographics*
220	*Radiology*
221	*Radiotherapy and Oncology*
222	*Respiratory Medicine*
223	*Seminars in Dialysis*
224	*Seminars in Nuclear Medicine*
225	*Seminars in Perinatology*
226	*Seminars in Respiratory and Critical Care Medicine*
227	*Seminars in Ultrasound, CT, and MR*
228	*Sexually Transmitted Diseases*
229	*Sexually Transmitted Infections*
230	*Social Science and Medicine*
231	*Southern Medical Journal*
232	*Spine*
233	*Statistical Methods in Medical Research*
234	*Statistics in Medicine*
235	*Stroke*
236	*Thorax*
237	*Thrombosis Research*
238	*Thyroid: official journal of the American Thyroid Association*
239	*Ultrasound in Obstetrics and Gynecology*
240	*Vaccine*
241	*World Neurosurgery*

A list of all recommended CUJ journals by subject can be found in Appendix F with the de-identified clinical usage data for each title and NLM ID. The list includes all the subjects assigned to a selected journal, even if one of the associated subjects was deemed beyond the scope of the project. Given that there are over 10,000 biomedical journals published presently, the new CUJ represents the essentials of clinical literature usage and may be useful as a tool for collection development by hospital libraries [13]. Hospital librarians recognize that their institutional services define what journals they need from this list. If the hospital does not deliver babies or transplant organs, titles in those subjects are not relevant for its collections.

With 21,428 journals included in Clarivate Analytics' Journal Citations Reports Master Journal List (2023) [14] and MEDLINE's growth from 2,300 to 5,288 indexed journals, the CUJ proposed list of 241 journals represents a core collection that is slightly less than MEDLINE's growth. The 241 selections are less than the 341 covered in five primary care review services, such as ACP Journal Club [15] or DynaMed [15, 16]; and it is less than the 250 medical journals included in the NEJM Journal Watch series [17].

## DISCUSSION

### Highest Clinical Impact Journals

Although the use of a journal-limited filter could eliminate relevant articles, CUJ's data-driven journal selections can also filter to the most highly clinically useful journals. The evidence-based selection added clinical journals that were missing from CCJ, including 14 titles that debuted on the list at #1 for clinical use in 15 subjects. (One journal appeared in 2 related subjects.) Notable new additions of contemporary relevance are the journals Clinical Infectious Diseases, Infection Control and Hospital Epidemiology, Journal of Emergency Medicine, Journal of Palliative Medicine and Vaccine. These highly used new additions are featured in Appendix G.

The resulting Clinically Useful Journals (CUJ) list reflects real-world evidence encompassing national discharge data, U.S. health goals for 2020, and actual journal usage by a wide variety of institutions and professionals across the country. Among the total usage of the over 1,600 journals analyzed, journals assigned to the new CUJ list accounted for about 85% of the usage. In addition to a one-click clinical limit, institutions may use the data-driven list to create customized searches in PubMed.gov for their institutional providers. Implementation will enable future evaluation of the scope and utility of the new list.

### Sustainability

The committee does not anticipate that the subjects covered by CUJ require frequent, regular review. At least every 15 years should be an adequate subject reevaluation schedule, although journals may warrant closer scrutiny.

The Evidence-based Usage Model process could be more automated. Some journal vendors are able to provide usage statistics to hospital libraries. As more hospital libraries automate their journal lists and receive electronic usage reports, data could be solicited from hospital libraries and furnished to future researchers to update the CUJ. This would automatically supply the JU counts needed, and Patient Driven Counts, some publicly available via AHRQ, won't be needed until subjects are reviewed again.

**Figure 2:**
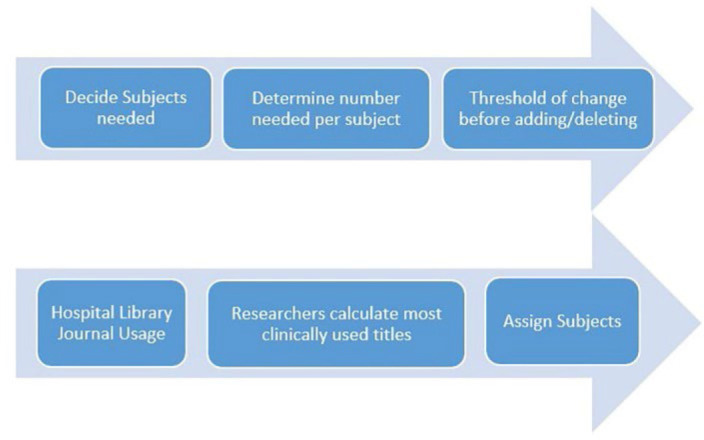
CUJ Automated Update Process

The CUJ Update Flow Chart (Figure 2) below illustrates an automation process for the CUJ.

With a more automated method to collect clinical journal usage and assign subjects to it, the process could be streamlined. It may be possible to create a ranked list with minimal manual handling.

## IMPLEMENTATION

To complete its charge, the committee made the following recommendations to MLA:

1. Accept report and transmit the newly created CUJ set listed here to NLM to fulfill the original request.
  - The CUJ data set will be extremely valuable for MLA members and other information professionals wishing to create searches within the new PubMed interface, as well as healthcare clinicians.
  - The implications for clinical practice are that customized searches may enhance search retrievals, improve efficiency and translate into improved patient care.
  - This filter can save clinicians' time, speed retrieval of clinically focused literature, and improve evidence-informed patient care.
  - With 75%-80% of the clinicians in the United States, Canada, and the United Kingdom searching PubMed [18], the potential increase in search efficiency from a new, clinically grounded journals filter is substantial. Time constraints present a major barrier to pursuing answers to clinical questions [18, 19]. The time to select documents from a list of search results could be significantly reduced with the new filter. Searching under time pressure, a constant for time-poor clinicians, may erode answer confidence and degrade decision quality [20]. The new Clinically Useful Journals list may speed up the search process by limiting the results to journals with documented clinical utility.
2. Promotion and Training
  - To increase acceptance and application of this new CUJ list, it is important that librarians, clinicians, stakeholders, and publishers understand how it was formulated. Its data-driven methodology differs from the consensus or survey approach used in the past to formulate publication lists. Its dependence on clinically used journals, and not just subscription numbers, identifies journal titles most likely to be applicable to healthcare organizations.
  - Clinicians and students need to learn that their searches can be limited to clinically well-used journals if they retrieve too many citations or are not seeing clinically useful results,
3. Evaluation and Review Cycle
  - Three years after implementation, researchers could obtain a snapshot study of usage of the CUJ filter. Comparative data from a full day PubMed query log [21, 22] could reveal CUJ usage.
  - Within 4-5 years after availability of the list, the call for journal usage could be distributed to Hospital Librarians and PALS libraries so another joint committee could re-evaluate the journals.
4. Subject Review
  - Consider reviewing the subjects within CUJ in 15 years.
  - Although the new CUJ list redefines the old CCJ list, it better reflects actual clinical applications. Missing heavily used clinical journals and contemporary subjects may have contributed to the previous low usage of CCJ as found in our study.
  - By using real-world data on clinical use of journals, the new list incorporates a Clinical Impact Factor in searches, potentially making PubMed more clinically relevant. Faster searches may result since the list incorporates highly used journals. Increased search efficiency may be a hidden bonus, resulting in clinician time saved.
  - The CUJ revised subject coverage is defined by JUs and PDCs, orienting the proposed list to sources of frequent healthcare spending. Coverage of heart diseases, diabetes, arthritis/rheumatism, cancer and mental disorders was expanded [23].

The new CUJ subject index list may identify high-priority journals to add to a library's collection, while the innovative data-driven techniques may reframe the discussion around evaluating the impact and utility of biomedical journals.

Over the course of the project, NLM/NCBI developed a new MEDLINE interface of PubMed.gov that was implemented in spring 2020. With that new interface, the sidebar filter option of the old Core Clinical Journal list was eliminated. The Advanced Search options of the new PubMed does offer sidebar limits. It will be up to the discretion of NLM to make an informed decision about the clinical utility of the new, updated Clinically Useful Journals (CUJ) data set and its implementation. A pilot test of the replacement filter could demonstrate its utility.

To enable its immediate application, the CUJ list has been translated into a PubMed search strategy and is available in Appendix H.

The potential to sort search results by “Best Match” in the current PubMed as one of several sorting options, may be enhanced by the CUJ subset. “Best Match” sorted results limited to the CUJ subset can limit results to the journals most likely to be clinically impactful. While the rigorously designed CUJ filter adds another step, it can strengthen the clinical relevance of a standard PubMed search by using a filter defined by clinical journal usage data. With over 80% of searchers' clicks occurring among the top 20 citations or first page of results returned [24], clinician search efficiency and satisfaction may be increased by applying the CUJ filter to “Best Match” results.

## LIMITATIONS

The Proportion method may be imperfect, but it was the most data-driven method to approximate the original ratio of CCJ journals to the total number of journals within MEDLINE. Although it included growth in non-clinical journals, over the decades some preclinical sciences became relevant to clinical queries. Medical Genetics is one example. Note that some journals retained NLM broad subjects beyond the scope of CUJ's new set of 80 subjects reflecting the relevance of an expanded focus.

The data collected on clinical use of journals were from North American library members of the Medical Library Association. Thus, the high use journals included English language titles, with many international journals represented. World-wide application of the journals selected might be limited in non-English speaking countries. Military, veterans, and indigenous populations may be underrepresented. Preclinical and animal subjects were omitted.

## CONCLUSIONS

Search efficiency is very important to librarians and clinicians alike. It is vital to have one universally available filter, whether logged in to a personal account or not, to automatically reduce retrieval to a focused, manageable set. By determining the most important subjects for clinical application using JUs and PDCs, and then selecting journals based on actual clinical usage, the new list is strongly oriented to the most clinically useful journals. Thus, the new CUJ list is optimized for clinical inquiries and offers evidence-selected journals to practitioners. Research shows that freely available PubMed/MEDLINE is frequently searched to answer clinical questions, second only to directly searching within journal issues [20, 25]. Having a CUJ filter to limit to those journals most frequently accessed for clinical queries would add a powerful tool.

Hospital librarians and physicians have applied essentially the same CCJ filter for 50 years. Research revealed gaps in journal coverage that are addressed by this newly updated, evidence-based CUJ filter. An updated list of journals reflecting actual clinical usage should result in search retrievals more applicable to real-world clinical questions and a broader range of healthcare practitioners. Implementing the new CUJ could benefit the entire healthcare community by encompassing high use journals for medical and mental conditions.

## Data Availability

Data associated with this article are available at https://figshare.com/ registered under DOI 10.6084/m9.figshare.21979832. This work is licensed under a Creative Commons Attribution 4.0 International License.

## SUPPLEMENTAL FILES

**Appendix A:** Ad Hoc Committee to review Core Clinical Journals; Charge, Cochairs, and Membership

**Appendix B:** Morning Report Blogs

**Appendix C:** Data Sources

**Appendix D:** Statistical Methods for Subject Analysis

**Appendix E:** Subject Analysis (expanded to show before/after)

**Appendix F:** Clinically Useful Journals (CUJ) by Subject with Usage Data and NLM ID

**Appendix G:** Fifteen Subjects with newly added Journals Ranked #1

**Appendix H:** Search strategy to retrieve the new CUJ

## References

[1] An abbreviated Index Medicus. JAMA. 1969 Dec 22;210(12):2272–3. https://jamanetwork.com/journals/jama/article-abstract/350909. 5395421

[2] Anonymous. Notice. Abridged Index Medicus. 1979 Jan;10(1) PMC197407 16017527

[3] Anonymous (2001) New look to PubMed's subset limit pull-down menu: Core Clinical Journals (used to be ‘AIM’). NLM Technical Bulletin, 2001 (319), 6.Bulletin, 2001 (319), 6.

[4] United States National Library of Medicine. Abridged Index Medicus (AIM or “Core Clinical”) Journal Titles. About MEDLINE® and PubMed®: The Resources Guide U.S. National Library of Medicine (NLM) [July 27, 2020 cited]. https://www.nlm.nih.gov/bsd/aim.html.

[5] Klein-Fedyshin M, Ketchum AM, Arnold RM, Fedyshin PJ. Evaluating the MEDLINE Core Clinical Journals filter: data-driven evidence assessing clinical utility. J Eval Clin Pract. 2014 Dec;20(6):837–43. 10.1111/jep.12190. 24904958

[6] Kaiser Permanente. Kaiser Permanente: Who We Are - Fast Facts [cited 2020]. https://about.kaiserpermanente.org/who-we-are/fast-facts.

[7] AHRQ. H-CUP National Inpatient Sample, Principal diagnoses. Overview of the National (Nationwide) Inpatient Sample (NIS). Healthcare Cost and Utilization Project (HCUP). Agency for Healthcare Research and Quality (AHRQ); Rockville, MD: [cited September 2022]. https://www.hcup-us.ahrq.gov/nisoverview.jsp.

[8] Doody's Enterprises Inc. Doody's Core Titles Oak Park, IL: Doody's Enterprises Inc. [cited 2019]. https://www.doody.com/dct/default.asp.

[9] U.S. Department of Health and Human Services. Healthy People 2020 Washington, DC: Office of Disease Prevention and Health Promotion. [cited 2019]. https://www.healthypeople.gov/2020/topics-objectives.

[10] Medscape. Medscape Topic Alerts WebMD LLC [cited 2019]. https://www.medscape.com/newsletters/topicalerts.

[11] U.S. National Library of Medicine (NLM). Broad Subject Terms for Indexed Journals [cited]. https://journal-reports.nlm.nih.gov/broad-subjects/#r.

[12] Centre for Science and Technology Studies. CWTS Journal Indicators: Methodology The Netherlands: Leiden University [cited]. https://www.journalindicators.com/methodology.

[13] Johnson R, Watkinson A, Mabe M. The STM Report: an overview of scientific and scholarly publishing. The Hague, The Netherlands: [cited 2019]. https://www.stm-assoc.org/2018_10_04_STM_Report_2018.pdf.

[14] Journal Citation Reports. Master Journals List. Clarivate Analytics; 2023. Downloaded 28 April 2023 from https://mjl.clarivate.com/collection-list-downloads.

[15] ACP Journal Club [Internet]. American College of Physicians, Inc. 2022. Available from: https://www.acpjournals.org/topic/category/journal-club.

[16] Alper BS, Hand JA, Elliott SG, Kinkade S, Hauan MJ, Onion DK, Sklar BM. How much effort is needed to keep up with the literature relevant for primary care? J Med Libr Assoc. 2004 Oct;92(4):429–37. https://www.ncbi.nlm.nih.gov/pubmed/15494758. 15494758 PMC521514

[17] NEJM Group. NEJM Journal Watch Waltham, MA: Massachusetts Medical Society [cited]. https://www.jwatch.org/about/products-and-services.

[18] Davies KS. Physicians and their use of information: a survey comparison between the United States, Canada, and the United Kingdom. J Med Libr Assoc. 2011 Jan;99(1):88–91. https://www.ncbi.nlm.nih.gov/pmc/articles/PMC3016665/. 21243061 10.3163/1536-5050.99.1.015 PMC3016665

[19] Brassil E, Gunn B, Shenoy AM, Blanchard R. Unanswered clinical questions: a survey of specialists and primary care providers. J Med Libr Assoc. 2017 Jan;105(1):4–11. https://www.ncbi.nlm.nih.gov/pmc/articles/PMC5234458/. 28096740 10.5195/jmla.2017.101 PMC5234458

[20] van der Vegt A, Zuccon G, Koopman B, Deacon A. How searching under time pressure impacts clinical decision making. J Med Libr Assoc. 2020 Oct 1;108(4):564–73. https://www.ncbi.nlm.nih.gov/pmc/articles/PMC7524617/. 33013213 10.5195/jmla.2020.915 PMC7524617

[21] Herskovic JR, Tanaka LY, Hersh W, Bernstam EV. A day in the life of PubMed: analysis of a typical day's query log. J Am Med Inform Assoc. 2007 Mar-Apr;14(2):212–20.z https://www.ncbi.nlm.nih.gov/pmc/articles/PMC2213463/. 17213501 10.1197/jamia.M2191 PMC2213463

[22] Mosa AS, Yoo I. A study on PubMed search tag usage pattern: association rule mining of a full-day PubMed query log. BMC Med Inform Decis Mak. 2013 Jan 9;13:8. https://bmcmedinformdecismak.biomedcentral.com/articles/10.1186/1472-6947-13-8. 23302604 10.1186/1472-6947-13-8 PMC3552776

[23] Biener AI, Decker SL, Rohde F. Source of Increased Health Care Spending in the United States. JAMA. 2019 Mar 26;321(12):1147. https://www.ncbi.nlm.nih.gov/pubmed/30912822. 30912822 10.1001/jama.2019.0679

[24] Fiorini N, Leaman R, Lipman D, Lu Z. How user intelligence is improving PubMed. Nature Biotechnology. 2018 36(10):937–45. https://www.nature.com/articles/nbt.4267. 10.1038/nbt.4267 30272675

[25] Dunn K, Marshall J, Wells A, Backus J. Examining the role of MEDLINE as a patient care information resource: an analysis of data from the Value of Libraries study. J Med Libr Assoc. 2017 105(4):336–46. https://www.ncbi.nlm.nih.gov/pmc/articles/PMC5624423/pdf/jmla-105-336.pdf. 28983197 10.5195/jmla.2017.87 PMC5624423

