# Supplementary material for: PubMed's core clinical journals filter: redesigned for contemporary clinical impact and utility
Source: J Med Libr Assoc. 2023 Jul 10;111(3):665–76. doi: 10.5195/jmla.2023.1631 (PMC10361554; doi:10.5195/jmla.2023.1631)
Supplement: Supplementary file 1 — Appendix A: Ad Hoc Committee to review Core Clinical Journals; Charge, Cochairs, and Membership [file jmla-111-3-665-s01.pdf]

Appendix A. Medical Library Association Ad Hoc Committee to Review Core Clinical Journals: Charge and Members

Committee Charge of the *MLA Ad Hoc Committee to Review Core Clinical Journals*

1. Produce an updated core clinical journal (CCJ) list for use in a revised PubMed filter to include journals that are of immediate interest to practicing healthcare individuals, and journals supporting hospital librarians in their efforts to provide access to the essential biomedical literature to their physician and healthcare providers.
2. Document the review process including any additional restrictions or requirements that are deemed important in order to provide a transparent process.
3. Recommend a periodic review process to maintain an updated list.

Committee Personnel

Cochair	Michele Klein-Fedyshin, HSLS, University of Pittsburgh, Pittsburgh, PA	kleinf@pitt.edu
Cochair	Andrea M. Ketchum HSLS, University of Pittsburgh (Emeritus), Pittsburgh, PA	ketchum@pitt.edu
Member	Julia M. Esparza, Louisiana State University. HSC Medical Center Library, Shreveport, LA	jespar@lsuhsc.edu
Member	Lisa H. Jacob, Advocate Health Care, Advocate Lutheran General Hospital, Park Ridge, IL (now retired)	lisa.jacob@advocatehealth.com
Member	Elizabeth J. Kiscaden, University of Iowa, Hardin Library for the Health Sciences, Iowa City, IA (Now at Creighton University)	ElizabethKiscaden@creighton.edu
Member	Kate Majewski, NLM MEDLARS Management Section, Bethesda, MD	majewsk@mail.nlm.nih.gov
Member	Kim Santoro, NLM Technical Services Division, Bethesda, MD	santork@mail.nlm.nih.gov
Ex Officio	David Gillikin, Chief, NLM Bibliographic Services Division, Bethesda, MD	gillikd@mail.nlm.nih.gov
Ex Officio	Mary Langman, MLA Director of Information Issues and Policy, Chicago, IL	langman@mail.mlahq.org
Ex Officio	Melissa Rethlefsen, MLA Board Liaison, Chicago, IL	mlrethlefsen@gmail.com
Consulting Statistician	Si-Fang Zhao, MS Biostatistics, University of Pittsburgh, Pittsburgh, PA	siz48@pitt.edu
