# Supplementary material for: PubMed's core clinical journals filter: redesigned for contemporary clinical impact and utility
Source: J Med Libr Assoc. 2023 Jul 10;111(3):665–76. doi: 10.5195/jmla.2023.1631 (PMC10361554; doi:10.5195/jmla.2023.1631)
Supplement: Supplementary file 2 — Appendix B: Morning Report Blogs [file jmla-111-3-665-s02.pdf]

Appendix B. Morning Report Blogs

Compiled in August 2015 - Searched Google using search term “internal medicine morning report”

1. *Georgetown University Department of Medicine http://medicine.georgetown.edu/residency/el_morningreportunknowns.htm - URL not active Currently at https://clinicalproblemsolving.com/blog/
2. *UCSF Internal Medicine Morning Report Blog (https://ucsfmed.wordpress.com/category/morning-report/ - URL still active.
3. *UWMC Morning Report http://blog.hsl.washington.edu/report/ - URL not active
4. *University of Utah Department of Pediatrics morning report blog (https://pcmcmorningreport.blogspot.com/ - URL still active
5. *Toronto General Hospital http://morningreporttgh.blogspot.com/- URL still Active
6. Health Sciences Library System, University of Pittsburgh, Morning Report Blog- Not online
7. Louisiana State University Health Science Library- Internal Medicine Morning Report- Not online
