# Supplementary material for: PubMed's core clinical journals filter: redesigned for contemporary clinical impact and utility
Source: J Med Libr Assoc. 2023 Jul 10;111(3):665–76. doi: 10.5195/jmla.2023.1631 (PMC10361554; doi:10.5195/jmla.2023.1631)
Supplement: Supplementary file 3 — Appendix C: Data Sources [file jmla-111-3-665-s03.pdf]

Appendix C. Data Sources

MLA Institutional Members	JU (Journal Use statistics)	Number of times specific journals were used during Morning Report (MR) as reported in the University of Pittsburgh and Louisiana State University at Shreveport health library studies.
Internet	JU	Online Morning Report blogs (Appendix 2)
MLA Members	JU	Kaiser Permanente 39-hospital and clinic system in 8 states and DC.
MLA Members	JU	Primary Access Libraries' journals (PALS) in small hospitals, clinics (tie-breaker)
Government data sources	PDC (Patient-Driven Counts	Healthcare Cost and Utilization Project (HCUP) of the Agency for Healthcare Research and Quality (AHRQ): National discharge statistics by diagnosis
Government	PDC	Healthy People 2020 Objectives from the U.S. Centers for Disease Control and Prevention(CDC)
Publisher	PDC	Medscape Topic frequency data for requested alerts
Publisher	PDC	Doody Core Titles' Specialties (with permission)
