# Supplementary material for: PubMed's core clinical journals filter: redesigned for contemporary clinical impact and utility
Source: J Med Libr Assoc. 2023 Jul 10;111(3):665–76. doi: 10.5195/jmla.2023.1631 (PMC10361554; doi:10.5195/jmla.2023.1631)
Supplement: Supplementary file 4 — Appendix D: Statistical Methods for Subject Analysis [file jmla-111-3-665-s04.pdf]

Appendix D. Statistical Methodology

<u>The Ad Hoc Committee decided to evaluate and update the CCJ **subject** coverage first</u>

The U.S. National Library of Medicine (NLM) applies broad subject headings to MEDLINE journals from a list of 125 Medical Subject Headings (MESH). Core Clinical Journals are indexed with these Broad Subject Headings. After the JU's and PDC's were gathered, the data was correlated by the statistician for all 125-subject headings. The correlation of JU and PDC data divided the Subject Headings into nine paired groups based on counts of Journal Usage and Patient Discharge contributions by our statistician, Sifang Zhao.

a. Methodology to divide the subject headings

> The scatter plot (Figure 1) shows the relationship between journal usage (JU) and patient-driven counts (PDC). The outlier near the JU-axis is the subject heading Medicine and the outlier on the bottom right is the subject heading Perinatology. The data is spread out, and there are widespread points on the Y and/or X axis. To address this issue, the data was divided into 4 groups initially (Figure 2). Table 1 below indicates rules. Then each group was divided into 3 more subgroups by the 25 and 75 percentiles (Figure 3). Based on the high and low of JU and PDC, there are Yes groups to keep, No groups to reject, and Middle/Maybe groups that needed discussion (Figure 4).

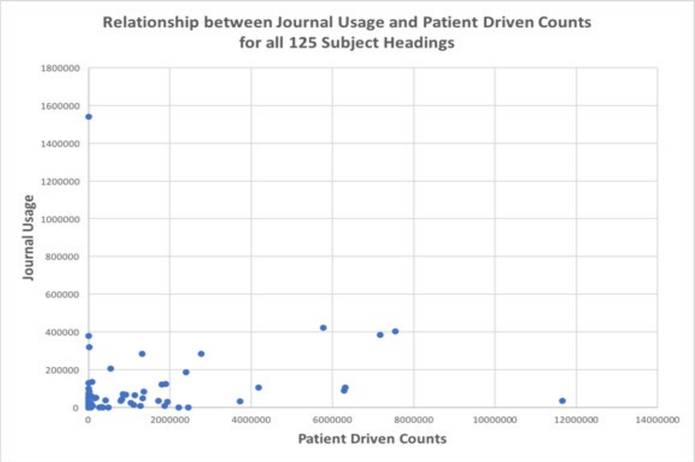

Figure 1. Scatter plot of JU's and PDC's

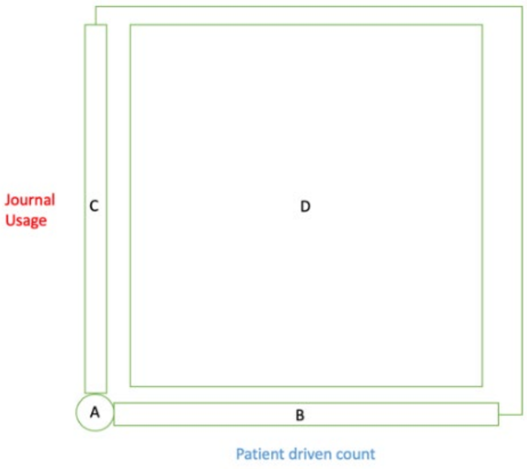

Figure 2. Data division into four groups

Table 1. Rules for data division

Group	Rules
A	JU=0 and PDC=0
B	JU=0
C	PDC=0
D	The rest

Figure 3. Groups subdivided by percentiles

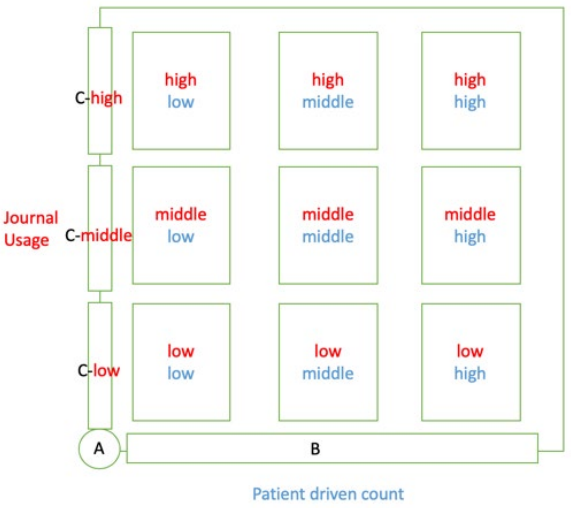

Figure 4. Subject decision structure

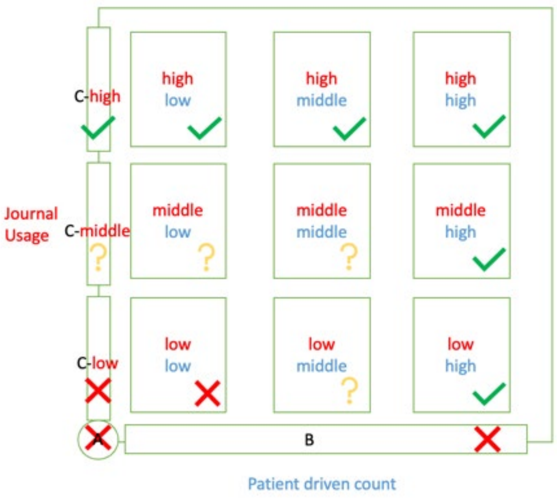

Thus, the formal Subject Selection Criteria for CCJ coverage that the committee used are:

- Selection Criteria 1: Keep all subjects with either high journal usage or high patient-driven counts unless JU<1000.

- Selection Criteria 2: Delete all subjects relating to animals

- ***Selection Criteria 3:*** *Delete other subjects with JU<1000*

- ***Selection Criteria 4:*** *Delete any remaining preclinical sciences (e.g., Biology, Cell Biology, Physics)*

We retained those subjects with high Journal Usage and high Patient Driven Counts (36); we eliminated those with both low JUs and low PDC's (30); those with middling JU's and PDC's were considered for retention. The committee agreed to the cut-off point of 1000 journal uses (JU) as a minimum**.** With this threshold, most of the JU=Middle and PDC=Middle (22) subjects were retained, and all of the JU=Middle and PDC=Low were kept (18). Among the JU=Middle and PDC=0, 4 subjects qualified for retention. All of the JU=Low and PDC=Middle (6) were deleted. Clinical journal usage dominated our decision-making. In all, 80 subjects were retained for CCJ coverage and 45 were deleted.

Journal Selection for the retained subjects

a. Deciding how many journals were needed per subject

Determining the number of journals per subject involved two data counts. Two methods determined how many journals were needed per subject. The first includes the Proportion method.

1. **Proportion Method**
   This method calculated what percentage of English language MEDLINE journals were included in the original Abridged Index Medicus in the conception year of 1970. This calculation was 100 Core journals/2300 English language MEDLINE journals =0.044. This percentage (0.044) was then applied to the English language MEDLINE journals indexed in 2018, or 5152, resulting in a total of 226. The committee decided on 222 journals designated for the new CCJ list. To determine how many were allocated to each subject, we determined each subject's percentage of clinical use and multiplied by 222. We calculated that a final total of 217 journals from the proportion method would result to cover the retained subjects at the same rate as the original Abridged Index Medicus.

   As an example, the subject *Medicine's* journal usage was 6.425% of the total. This percentage was multiplied by the 222 target to give 14 needed journals to cover this largest category.

2. **Parallel Analysis Method**
   On the recommendation that the process needed a parallel analysis, we contacted Sifang Zhao, our previous statistician. Parallel Analysis is a method to determine components to keep in a *Principal Component Analysis* or *factor analysis*. Its purpose is to use a few specified factors to describe the relationship between many variables. It studies the relationship between variables, explores the basic data structures, and uses components to express data features. In our analysis, we used the four components or factors Journal Usage, Subject Frequency, Patient Discharge Counts and Elsevier's Source Normalized Impact per Paper (SNIP) score, a citation metric normalized across subject fields to avoid bias. The 2017 SNIP score for each journal was listed under each broad subject heading. SNIP scores are ranked from high to low within each

subject heading. All subject headings were divided into 5 groups based on the current number of journals in each subject.

As an example, Figure 5 suggests adding two journals for the Subject Headings in Group 1.

Figure 5. Parallel analysis example

```
Using eigendecomposition of correlation matrix.
Computing: 10%  20%  30%  40%  50%  60%  70%  80%  90%  100%


Results of Horn's Parallel Analysis for component retention
1200 iterations, using the mean estimate

--------------------------------------------------
Component   Adjusted    Unadjusted    Estimated
            Eigenvalue  Eigenvalue    Bias
--------------------------------------------------
1          17.634047   25.987254      8.353207
2           3.660255   10.393909      6.733653
--------------------------------------------------

Adjusted eigenvalues > 1 indicate dimensions to retain.
(2 components retained)
```

Individual journals' total scores were merged into the Subject Headings using the journal's NLM-assigned Broad Subject Headings. At the committee’s meeting, PowerPoints displayed the calculations and recommended increments by group.

3. Cumulating the two methods
   The two methods of calculating journals needed per subject were merged giving a resulting maximum number of journals recommended for each subject. In no cases was the maximum number of journals allowed per subject exceeded. In several cases, fewer journal per subject were selected because of insufficient clinical usage. This cumulation resulted in a maximum of 254 journals for the new CCJ.

   This number of journals is less than the 341 covered in five primary care review services [9] and compares to the over 250 medical journals included in the NEJM Journal Watch series [10]. Since this is the first update of both subjects and journals in 50 years, it is unlikely that the number of subjects or journals would increase significantly in the next review.
