# Supplementary material for: PubMed's core clinical journals filter: redesigned for contemporary clinical impact and utility
Source: J Med Libr Assoc. 2023 Jul 10;111(3):665–76. doi: 10.5195/jmla.2023.1631 (PMC10361554; doi:10.5195/jmla.2023.1631)
Supplement: Supplementary file 5 — Appendix E: Subject Analysis (expanded to show before/after) [file jmla-111-3-665-s05.pdf]

Appendix E

Complete Subject Analysis

<u>What's new? These 33 Clinical Subjects previously had no journal coverage</u>

1. Acquired Immunodeficiency Syndrome
2. Microbiology
3. Sexually Transmitted Diseases
4. Behavioral Sciences
5. Psychology
6. Psychopharmacology
7. Psychophysiology (Pain)
8. Substance-Related Disorders
9. Embryology
10. Reproductive Medicine
11. Women's Health
12. Palliative Care
13. Health Services
14. Health Services Research
15. Transplantation
16. Podiatry
17. Sports Medicine
18. Diagnostic Imaging
19. Nuclear Medicine
20. Radiotherapy
21. Anti-Infective Agents
22. Antineoplastic Agents
23. Social Sciences
24. Biomedical Engineering
25. Medical Informatics
26. Nephrology
27. Physiology
28. Complementary Therapies
29. Biochemistry
30. Genetics, Medical
31. Molecular Biology
32. Epidemiology
33. Statistics as Topic

<u>These Subjects were previously covered in CCJ.</u> (Depth may be new)

1. Allergy & Immunology
2. Dermatology
3. Rheumatology
4. Psychiatry
5. Brain
6. Neurology
7. Neurosurgery
8. Gynecology
9. Obstetrics
10. Pediatrics
11. Perinatology
12. Dentistry
13. Ophthalmology
14. Otolaryngology
15. Gastroenterology
16. Geriatrics
17. Internal Medicine
18. Medicine
19. Primary Health Care (Family Practice)
20. Hospitals
21. General Surgery
22. Anesthesiology
23. Emergency Medicine
24. Occupational Medicine
25. Traumatology
26. Orthopedics
27. Physical Medicine & Rehabilitation
28. Radiology
29. Cardiology
30. Critical Care
31. Pulmonary Medicine
32. Vascular Diseases
33. Communicable Diseases
34. Drug Therapy
35. Hematology
36. Neoplasms
37. Pharmacology
38. Therapeutics
39. Education
40. Endocrinology
41. Metabolism
42. Urology
43. Nursing
44. Nutritional Sciences
45. Pathology
46. Public Health
47. Environmental Health
