# Supplementary material for: PubMed's core clinical journals filter: redesigned for contemporary clinical impact and utility
Source: J Med Libr Assoc. 2023 Jul 10;111(3):665–76. doi: 10.5195/jmla.2023.1631 (PMC10361554; doi:10.5195/jmla.2023.1631)
Supplement: Supplementary file 6 — Appendix F: Clinically Useful Journals (CUJ) by Subject with Usage Data and NLM ID [file jmla-111-3-665-s06.pdf]

Appendix F. Clinically Useful Journals (CUJ) by Subject with Usage Data and NLM ID

JOURNAL TITLE	SUBJECT HEADING	CLINICAL USE	NLMID
Journal of Acquired Immune Deficiency Syndromes: JAIDS	Acquired Immunodeficiency Syndromes	3297	100892005
Vaccine	Allergy and Immunology	8454	8406899
Annals of Allergy, Asthma, and Immunology	Allergy and Immunology	5154	9503580
Journal of Allergy and Clinical Immunology	Allergy and Immunology	2656	1275002
Allergy	Allergy and Immunology	2183	7804028
Autoimmunity Reviews	Allergy and Immunology	1900	101128967
British Journal of Anaesthesia	Anesthesiology	25139	0372541
Anesthesia and Analgesia	Anesthesiology	18613	1310650
International Journal of Antimicrobial Agents	Anti-Infective Agents	2647	9111860
Cancer Treatment Reviews	Antineoplastic Agents, Neoplasms	1794	7502030
Addictive Behaviors	Behavioral Sciences, Substance Related Disorders	1986	7603486
Epilepsy and Behavior	Behavioral Sciences, Neurology	1912	100892858
Clinical Biochemistry	Biochemistry	1396	0133660
Molecular Genetics and Metabolism	Biochemistry, Molecular Biology, Metabolism	1206	9805456
Clinical Biomechanics	Biomedical Engineering, Physiology	1297	8611877
Stroke	Brain, Vascular Diseases	76951	0235266
Brain: a journal of neurology	Brain, Neurology	5123	0372537
Epilepsia	Brain, Neurology	4432	2983306R
Circulation	Cardiology, Vascular Diseases	276579	0147763
Journal of the American College of Cardiology	Cardiology	23299	8301365
European Heart Journal	Cardiology	16561	8006263
Heart (British Cardiac Society)	Cardiology	16168	9602087
International Journal of Cardiology	Cardiology	5249	8200291
Europace	Cardiology, Physiology	5105	100883649
Journal of Thoracic and Cardiovascular Surgery	Cardiology, Pulmonary Medicine, Vascular Diseases, General Surgery	4072	0376343
European Journal of Cardiothoracic Surgery	Cardiology, Pulmonary Medicine, General Surgery	3434	8804069
Catheterization and Cardiovascular Interventions	Cardiology	3301	100884139
Heart Rhythm	Cardiology	2561	101200317
European Journal of Heart Failure	Cardiology	2361	100887595
Current Opinion in Cardiology	Cardiology	1921	8608087
Journal of Cardiac Failure	Cardiology	1744	9442138
American Heart Journal	Cardiology	1515	0370465

Clinical Infectious Diseases	Communicable Diseases	98106	9203213
Infection Control and Hospital Epidemiology	Communicable Diseases, Epidemiology, Hospitals, Nursing	14054	8804099
Journal of Infectious Diseases	Communicable Diseases	8628	0413675
Pediatric Infectious Disease Journal	Communicable Diseases, Pediatrics	6875	8701858
Journal of Hospital Infection	Communicable Diseases, Hospitals	2251	8007166
Journal of Infection	Communicable Diseases	1531	7908424
Diagnostic Microbiology and Infectious Disease	Communicable Diseases, Microbiology	1492	8305899
Journal of Alternative and Complimentary Medicine	Complimentary Therapies	1662	9508124
Critical Care Medicine	Critical Care	17453	0355501
American Journal of Respiratory and Critical Care Medicine	Critical Care, Pulmonary Medicine	13592	9421642
AACN Advanced Critical Care	Critical Care, Nursing	3297	101269322
Seminars in Respiratory and Critical Care Medicine	Critical Care, Pulmonary Medicine	2565	9431858
Journal of Oral and Maxillofacial Surgery	Dentistry, General Surgery	3364	8206428
Oral Surgery, Oral Medicine, Oral Pathology and Oral Radiology	Dentistry, General Surgery	1529	9508562
JAMA Dermatology	Dermatology	65892	101589530
British Journal of Dermatology	Dermatology	7770	0004041
Pediatric Dermatology	Dermatology, Pediatrics	4861	8406799
Journal of Investigative Dermatology	Dermatology	4240	0426720
Ultrasound in Obstetrics and Gynecology	Diagnostic Imaging, Gynecology, Obstetrics	11090	9108340
Seminars in Ultrasound, CT, and MR	Diagnostic Imaging	1773	8504689
Gastrointestinal Endoscopy	Diagnostic Imaging, Therapeutics, Gastroenterology	1566	0010505
Medical Letter on Drugs and Therapeutics	Drug Therapy, Pharmacology, Therapeutics	25778	2985240R
Clinical Pharmacology and Therapeutics	Drug Therapy, Pharmacology	5247	0372741
Pharmacoepidemiology and Drug Safety	Drug Therapy, Epidemiology	4782	9208369
Annals of Pharmacotherapy	Drug Therapy, Pharmacology	4316	9203131
Clinical Therapeutics	Drug Therapy, Therapeutics	3634	7706726
Drugs	Drug Therapy, Pharmacology	2108	7600076
Academic Medicine	Education	2448	8904605
Early Human Development	Embryology, Pediatrics, Perinatology	1706	7708381
Journal of Emergency Medicine	Emergency Medicine	7753	8412174
Academic Emergency Medicine	Emergency Medicine	7488	9418450
Annals of Emergency Medicine	Emergency Medicine	2971	8002646
Journal of Clinical Endocrinology and Metabolism	Endocrinology, Metabolism	32281	0375362
Diabetes Care	Endocrinology	11403	7805975
Diabetes Research and Clinical Practice	Endocrinology	2561	8508335
Thyroid: official journal of the American Thyroid Association	Endocrinology	2531	9104317

Journal of Occupational and Environmental Medicine	Environmental Health, Occupational Medicine	2500	9504688
American Journal of Epidemiology	Epidemiology	16089	7910653
Infection Control and Hospital Epidemiology	Epidemiology, Hospitals, Communicable Diseases, Nursing	14054	8804099
Pharmacoepidemiology and Drug Safety	Epidemiology, Drug Therapy	4782	9208369
American Journal of Gastroenterology	Gastroenterology	23784	0421030
Gut	Gastroenterology	16260	2985108R
Gastroenterology	Gastroenterology	11514	0374630
Hepatology	Gastroenterology	9460	8302946
Clinical Gastroenterology and Hepatology	Gastroenterology	7065	101160775
Journal of Clinical Gastroenterology	Gastroenterology	4685	7910017
Diseases of the Colon and Rectum	Gastroenterology	4654	0372764
Journal of Pediatric Gastroenterology and Nutrition	Gastroenterology, Pediatrics, Nutritional Sciences	4261	8211545
Digestive Diseases and Sciences	Gastroenterology	4241	7902782
Journal of Hepatology	Gastroenterology	2564	8503886
Liver Transplantation	Gastroenterology, Transplantation	2552	100909185
Current Opinion in Gastroenterology	Gastroenterology	2145	8506887
Gastrointestinal Endoscopy	Gastroenterology, Diagnostic Imaging, Therapeutics	1566	0010505
Journal of Bone and Joint Surgery. American Volume	General Surgery, Orthopedics	38467	0014030
JAMA Surgery	General Surgery	21290	101589553
Plastic and Reconstructive Surgery	General Surgery	16046	1306050
American Journal of Surgical Pathology	General Surgery, Pathology	15187	7707904
Otolaryngology-Head and Neck Surgery	General Surgery, Otolaryngology	13900	8508176
Annals of Surgery	General Surgery	11029	0372354
Journal of Vascular Surgery	General Surgery, Vascular Diseases	9848	8407742
Journal of Foot and Ankle Surgery	General Surgery, Orthopedics,	9617	9308427
Annals of Thoracic Surgery	General Surgery, Pulmonary Medicine	8568	15030100R
Journal of Hand Surgery	General Surgery, Traumatology	8218	7609631
Journal of Trauma and Acute Care Surgery	General Surgery, Traumatology	7990	101570622
Journal of Pediatric Surgery	General Surgery, Pediatrics	6328	0052631
Head & Neck	General Surgery, Neoplasms	4100	8902541
Journal of Thoracic and Cardiovascular Surgery	General Surgery, Vascular Diseases, Pulmonary Medicine, Cardiology	4072	0376343
Clinics in Podiatric Medicine and Surgery	General Surgery, Podiatry	3995	8604974
European Journal of Cardiothoracic Surgery	General Surgery, Cardiology, Pulmonary Medicine	3434	8804069
Annals of Surgical Oncology	General Surgery, Neoplasms	3380	9420840
Journal of Oral and Maxillofacial Surgery	General Surgery, Dentistry	3364	8206428

European Journal of Surgical Oncology	General Surgery, Neoplasms	2297	8504356
Journal of Surgical Oncology	General Surgery, Neoplasms	2514	0222643
Oral Surgery, Oral Medicine, Oral Pathology and Oral Radiology	General Surgery, Dentistry	1529	9508562
Journal of Medical Genetics	Genetics, Medical	9520	2985087R
American Journal of Medical Genetics Part A	Genetics, Medical	8936	101235741
Journal of the American Geriatrics Society	Geriatrics	15628	7503062
Age and Ageing	Geriatrics	2400	0375655
Obstetrics and Gynecology	Gynecology, Obstetrics	130036	0401101
Gynecologic Oncology	Gynecology, Neoplasms	13078	0365304
Ultrasound in Obstetrics and Gynecology	Gynecology, Obstetrics, Diagnostic Imaging	11090	9108340
Obstetrical and Gynecological Survey	Gynecology, Obstetrics	7683	0401007
American Journal of Obstetrics and Gynecology	Gynecology, Obstetrics	7237	0370476
Clinical Obstetrics and Gynecology	Gynecology, Obstetrics,	6227	0070014
BJOG: an international journal of obstetrics and gynaecology	Gynecology, Obstetrics	5863	100935741
Journal of Obstetric, Gynecologic, and Neonatal Nursing: JOGNN	Gynecology, Perinatology, Nursing, Obstetrics	5048	8503123
International Urogynecology Journal	Gynecology, Urology	2066	101567041
Social Science and Medicine	Health Services, Social Sciences	5906	8303205
Medical Care	Health Services	5117	0230027
Health Affairs	Health Services Research, Public Health	89255	8303128
Journal for Healthcare Quality	Health Services Research	1250	9202994
Blood	Hematology	37489	7603509
British Journal of Haematology	Hematology	8208	0372544
American Journal of Hematology	Hematology	5895	7610369
Leukemia	Hematology, Neoplasms	4936	8704895
Journal of Thrombosis and Haemostasis	Hematology	3185	101170508
Journal of Pediatric Hematology/Oncology	Hematology, Neoplasms, Pediatrics	2512	9505928
Infection Control and Hospital Epidemiology	Hospitals, Communicable Diseases, Epidemiology, Nursing	14054	8804099
Journal of Hospital Medicine	Hospitals	6991	101271025
Journal of Hospital Infection	Hospitals, Communicable Diseases	2251	8007166
JAMA Internal Medicine incl Archives	Internal Medicine	108888	101589534
Annals of Internal Medicine	Internal Medicine	34446	0372351
Journal of General Internal Medicine	Internal Medicine	8861	8605834
European Journal of Internal Medicine	Internal Medicine	1542	9003220
Journal of Internal Medicine	Internal Medicine	932	8904841
Journal of the American Medical Informatics Association: JAMIA	Medical Informatics	8851	9430800
Computers, Informatics, Nursing: CIN	Medical Informatics, Nursing	1988	101141667

New England Journal of Medicine	Medicine	1039625	0255562
JAMA	Medicine	314455	7501160
BMJ (Clinical research ed.)	Medicine	121140	8900488
Lancet	Medicine	49836	2985213R
Postgraduate Medical Journal	Medicine	5448	0234135
QJM: monthly journal of the Association of Physicians	Medicine	3876	9438285
American Journal of the Medical Sciences	Medicine	2984	0370506
Journal of the American Medical Directors Association	Medicine	2343	100893243
Medicine (Baltimore)	Medicine	1824	2985248R
American Journal of Medicine	Medicine	1674	0267200
British Medical Bulletin	Medicine	1541	0376542
Southern Medical Journal	Medicine	1424	0404522
International Journal of Clinical Practice	Medicine	1153	9712381
Medical Clinics of North America	Medicine	1054	2985236R
CMAJ: Canadian Medical Association Journal	Medicine	647	9711805
Journal of Clinical Endocrinology and Metabolism	Metabolism, Endocrinology	32281	0375362
Obesity	Metabolism, Physiology	8789	101264860
International Journal of Obesity	Metabolism	5202	101256108
Molecular Genetics and Metabolism	Metabolism, Biochemistry, Molecular Biology	1206	9805456
Diagnostic Microbiology and Infectious Disease	Microbiology, Communicable Diseases	1492	8305899
Molecular Genetics and Metabolism	Molecular Biology, Metabolism, Biochemistry	1206	9805456
Journal of Clinical Oncology	Neoplasms	144611	8309333
Cancer	Neoplasms	23381	0374236
Annals of Oncology	Neoplasms	22325	9007735
Journal of the National Cancer Institute	Neoplasms	19111	7503089
Gynecologic Oncology	Neoplasms, Gynecology	13078	0365304
International Journal of Radiation Oncology, Biology, Physics	Neoplasms, Radiology, Radiotherapy	11423	7603616
British Journal of Cancer	Neoplasms	6533	0370635
CA: a Cancer Journal for Clinicians	Neoplasms	5931	0370647
Leukemia	Neoplasms, Hematology	4936	8704895
Head & Neck	Neoplasms, General Surgery	4100	8902541
European Journal of Cancer	Neoplasms	3612	9005373
International Journal of Cancer	Neoplasms	3427	0042124
Annals of Surgical Oncology	Neoplasms, General Surgery	3380	9420840
Journal of Surgical Oncology	Neoplasms, General Surgery	2514	0222643
Journal of Pediatric Hematology/Oncology	Neoplasms, Hematology, Pediatrics	2512	9505928

Radiotherapy and Oncology	Neoplasms, Radiotherapy	2468	8407192
European Journal of Surgical Oncology	Neoplasms, General Surgery	2297	8504356
Cancer Treatment Reviews	Neoplasms, Antineoplastic Agents	1794	7502030
Kidney International	Nephrology	16678	0323470
Nephrology, Dialysis, Transplantation	Nephrology, Transplantation	10374	8706402
American Journal of Kidney Diseases	Nephrology	2126	8110075
Current Opinion in Nephrology and Hypertension.	Nephrology, Vascular Diseases	1681	9303753
Seminars in Dialysis	Nephrology	1148	8911629
Journal of Neurology, Neurosurgery, and Psychiatry	Neurology, Psychiatry, Neurosurgery	23682	2985191R
JAMA Neurology	Neurology	16800	101589536
Neurology	Neurology	14316	0401060
Pain	Neurology, Psychophysiology	8031	7508686
Pain Medicine	Neurology, Psychophysiology, Palliative Care	5916	100894201
Brain: a journal of neurology	Neurology, Brain	5123	0372537
Epilepsia	Neurology, Brain	4432	2983306R
Annals of Neurology	Neurology	4119	7707449
Movement Disorders	Neurology	3862	8610688
Headache	Neurology, Psychophysiology	3625	2985091R
Muscle and Nerve	Neurology, Physiology	3601	7803146
Journal of Clinical Neuroscience	Neurology	3202	9433352
Epilepsy and Behavior	Neurology, Behavioral Sciences	1912	100892858
Journal of Neurology, Neurosurgery, and Psychiatry	Neurosurgery, Neurology, Psychiatry	23682	2985191R
Neurosurgery	Neurosurgery	10838	7802914
World Neurosurgery	Neurosurgery	2141	101528275
Seminars in Nuclear Medicine	Nuclear Medicine	1517	1264464
European Journal of Nuclear Medicine and Molecular Imaging	Nuclear Medicine, Physics	664	101140988
Infection Control and Hospital Epidemiology	Nursing, Hospitals, Communicable Diseases, Epidemiology	14054	8804099
Journal of Nursing Administration	Nursing	9765	1263116
American Journal of Nursing -AJN	Nursing	8879	0372646
Journal of Advanced Nursing	Nursing	7297	7609811
Nursing	Nursing	6423	7600137
Patient Education and Counseling	Nursing	5169	8406280
Journal of Obstetric, Gynecologic, and Neonatal Nursing: JOGNN	Nursing, Obstetrics, Gynecology, Perinatology	5048	8503123
AACN Advanced Critical Care	Nursing, Critical Care	3297	101269322
Journal of Midwifery and Women's Health	Nursing, Obstetrics, Women's Health	2482	100909407
Computers, Informatics, Nursing: CIN	Nursing, Medical Informatics	1988	101141667

Breastfeeding Medicine	Nutritional Sciences	4424	101260777
Journal of Pediatric Gastroenterology and Nutrition	Nutritional Sciences, Gastroenterology, Pediatrics	4261	8211545
JPEN. Journal of Parenteral and Enteral Nutrition	Nutritional Sciences	2893	7804134
Obstetrics and Gynecology	Obstetrics, Gynecology	130036	0401101
Ultrasound in Obstetrics and Gynecology	Obstetrics, Gynecology, Diagnostic Imaging	11090	9108340
Obstetrical and Gynecological Survey	Obstetrics, Gynecology	7683	0401007
American Journal of Obstetrics and Gynecology	Obstetrics, Gynecology	7237	0370476
Clinical Obstetrics and Gynecology	Obstetrics, Gynecology	6227	0070014
BJOG: an international journal of obstetrics and gynaecology	Obstetrics, Gynecology	5863	100935741
Journal of Obstetric, Gynecologic, and Neonatal Nursing: JOGNN	Obstetrics, Gynecology, Perinatology, Nursing	5048	8503123
Journal of Midwifery and Women's Health	Obstetrics, Nursing, Women's Health	2482	100909407
Journal of Occupational and Environmental Medicine	Occupational Medicine, Environmental Health	2500	9504688
JAMA Ophthalmology	Ophthalmology	18363	101589539
British Journal of Ophthalmology	Ophthalmology	11879	0421041
Journal of Bone and Joint Surgery. American Volume	Orthopedics, General Surgery	38467	0014030
Spine	Orthopedics	21051	7610646
Journal of Orthopaedic and Sports Physical Therapy	Orthopedics, Physical and Rehabilitation Medicine, Sports Medicine	10077	7908150
Journal of Foot and Ankle Surgery	Orthopedics, General Surgery	9617	9308427
Arthroscopy: Journal of Arthroscopic and Related Surgery	Orthopedics	8974	8506498
Journal of Orthopaedic Trauma	Orthopedics, Traumatology	8034	8807705
Journal of Shoulder and Elbow Surgery	Orthopedics, General Surgery	6414	9206499
Journal of Pediatric Orthopedics	Orthopedics, Pediatrics	4928	8109053
Otolaryngology-Head and Neck Surgery	Otolaryngology, General Surgery	13900	8508176
JAMA Otolaryngology-- Head & Neck surgery	Otolaryngology	13509	101589542
Laryngoscope	Otolaryngology	10080	8607378
Journal of Palliative Medicine	Palliative Care	13369	9808462
Journal of Pain and Symptom Management	Palliative Care, Psychophysiology, Therapeutics	6730	8605836
Pain Medicine	Palliative Care, Neurology, Psychophysiology	5916	100894201
Modern Pathology	Pathology	18210	8806605
American Journal of Surgical Pathology	Pathology, General Surgery	15187	7707904
Journal of Clinical Pathology	Pathology	8751	0376601
Human Pathology	Pathology	2814	9421547
Pediatrics	Pediatrics	208720	0376422
Archives of Disease in Childhood	Pediatrics	14955	0372434
JAMA Pediatrics	Pediatrics	24761	101589544

Pediatric Infectious Disease Journal	Pediatrics, Communicable Diseases	6875	8701858
Archives of Disease in Childhood. Fetal and Neonatal ed.	Pediatrics, Perinatology	6640	9501297
Journal of Pediatric Surgery	Pediatrics, General Surgery	6328	0052631
Journal of Pediatric Orthopedics	Pediatrics, Orthopedics	4928	8109053
Pediatric Dermatology	Pediatrics, Dermatology	4861	8406799
Current Opinion in Pediatrics	Pediatrics	4536	9000850
Journal of Pediatric Gastroenterology and Nutrition	Pediatrics, Nutritional Sciences, Gastroenterology	4261	8211545
Journal of Pediatric Hematology/Oncology	Pediatrics, Hematology, Neoplasms	2512	9505928
Early Human Development	Pediatrics, Perinatology, Embryology	1706	7708381
Journal of Perinatology	Perinatology	10980	8501884
Archives of Disease in Childhood. Fetal and Neonatal ed.	Perinatology, Pediatrics	6640	9501297
Journal of Obstetric, Gynecologic, and Neonatal Nursing: JOGNN	Perinatology, Nursing, Obstetrics, Gynecology	5048	8503123
Seminars in Perinatology	Perinatology	2712	7801132
Early Human Development	Perinatology, Embryology, Pediatrics	1706	7708381
Medical Letter on Drugs and Therapeutics	Pharmacology, Therapeutics, Drug Therapy	25778	2985240R
Clinical Pharmacology and Therapeutics	Pharmacology, Drug Therapy	5247	0372741
Annals of Pharmacotherapy	Pharmacology, Drug Therapy	4316	9203131
Journal of Orthopaedic and Sports Physical Therapy	Physical and Rehabilitation Medicine, Orthopedics, Sports Medicine	10077	7908150
Archives of Physical Medicine and Rehabilitation	Physical and Rehabilitation Medicine	7291	2985158R
Obesity	Physiology, Metabolism	8789	101264860
Europace	Physiology, Cardiology	5105	100883649
Muscle and Nerve	Physiology, Neurology	3601	7803146
Clinical Biomechanics	Physiology, Biomedical Engineering	1297	8611877
Clinics in Podiatric Medicine and Surgery	Podiatry. General Surgery	3995	8604974
American Family Physician	Primary Health Care	5562	1272646
Primary Care: Clinics in Office Practice	Primary Health Care	846	0430463
Journal of Neurology, Neurosurgery, and Psychiatry	Psychiatry, Neurosurgery, Neurology,	23682	2985191R
JAMA Psychiatry	Psychiatry, Psychology	15062	101589550
American Journal of Psychiatry	Psychiatry	14761	0370512
Biological Psychiatry	Psychiatry, Psychophysiology	3612	0213264
Psychiatric Services	Psychiatry	3388	950283
Journal of Clinical Psychology	Psychology	1199	0217132
Journal of Clinical Psychopharmacology	Psychopharmacology	3894	8109496
Journal of Psychopharmacology	Psychopharmacology	222	8907828

Pain	Psychophysiology, Neurology	8031	7508686
Journal of Pain and Symptom Management	Psychophysiology, Therapeutics, Palliative Care	6730	8605836
Pain Medicine	Psychophysiology, Palliative Care, Neurology	5916	100894201
Headache: The Journal of Head and Face Pain	Psychophysiology, Neurology	3625	2985091R
Biological Psychiatry	Psychophysiology, Psychiatry	3612	0213264
Health Affairs	Public Health, Health Services Research	89255	8303128
American Journal of Preventive Medicine	Public Health	7393	8704773
Preventive Medicine	Public Health	3707	0322116
Chest	Pulmonary Medicine	42008	0231335
American Journal of Respiratory and Critical Care Medicine	Pulmonary Medicine, Critical Care	13592	9421642
Thorax	Pulmonary Medicine	13447	0417353
Annals of Thoracic Surgery	Pulmonary Medicine, General Surgery	8568	15030100R
Journal of Thoracic and Cardiovascular Surgery	Pulmonary Medicine, Cardiology, General Surgery, Vascular Diseases	4072	0376343
European Journal of Cardiothoracic Surgery	Pulmonary Medicine, General Surgery, Cardiology	3434	8804069
Seminars in Respiratory and Critical Care Medicine	Pulmonary Medicine, Critical Care	2565	9431858
Respiratory Medicine	Pulmonary Medicine	2131	8908438
AJR. American Journal of Roentgenology	Radiology	183579	7708173
Radiology	Radiology	104676	0401260
Radiographics	Radiology	66916	8302501
International Journal of Radiation Oncology, Biology, Physics	Radiology, Radiotherapy, Neoplasms	11423	7603616
European Journal of Radiology	Radiology	4278	8106411
Journal of Vascular and Interventional Radiology: JVIR	Radiology, Vascular Diseases	3808	9203369
International Journal of Radiation Oncology, Biology, Physics	Radiotherapy, Neoplasms, Radiology	11423	7603616
Radiotherapy and Oncology	Radiotherapy, Neoplasms	2468	8407192
Fertility and Sterility	Reproductive Medicine	12927	0372772
Human Reproduction	Reproductive Medicine	9075	8701199
Annals of the Rheumatic Diseases	Rheumatology	16720	0372355
Arthritis & Rheumatology	Rheumatology	10702	101623795
Arthritis Care & Research	Rheumatology	5123	101518086
Current Opinion in Rheumatology	Rheumatology	4578	9000851
Best Practice and Research. Clinical Rheumatology	Rheumatology	2119	101121149
Sexually Transmitted Infections	Sexually Transmitted Diseases	2442	9805554
Sexually Transmitted Diseases	Sexually Transmitted Diseases	455	7705941
Social Science and Medicine	Social Sciences, Health Services	5906	8303205
American Journal of Sports Medicine	Sports Medicine	19380	7609541

Journal of Orthopaedic and Sports Physical Therapy	Sports Medicine, Physical and Rehabilitation Medicine, Orthopedics	10077	7908150
Statistics in Medicine	Statistics as Topic	5580	8215016
Statistical Methods in Medical Research	Statistics as Topic	190	9212457
Drug and Alcohol Dependence	Substance-Related Disorders	3516	7513587
Journal of Substance Abuse Treatment	Substance-Related Disorders	2481	8500909
Addictive Behaviors	Substance Related Disorders, Behavioral Sciences	1986	7603486
Medical Letter on Drugs and Therapeutics	Therapeutics, Drug Therapy, Pharmacology	25778	2985240R
Journal of Pain and Symptom Management	Therapeutics, Palliative Care, Psychophysiology	6730	8605836
Clinical Therapeutics	Therapeutics, Drug Therapy	3634	7706726
Gastrointestinal Endoscopy	Therapeutics, Gastroenterology, Diagnostic Imaging	1566	0010505
Nephrology, Dialysis, Transplantation	Transplantation, Nephrology	10374	8706402
Bone Marrow Transplantation	Transplantation	5060	8702459
Liver Transplantation	Transplantation, Gastroenterology	2552	100909185
Journal of Hand Surgery	Traumatology, General Surgery	8218	7609631
Journal of Orthopaedic Trauma	Traumatology, Orthopedics	8034	8807705
Journal of Trauma and Acute Care Surgery	Traumatology, General Surgery	7990	101570622
Journal of Urology	Urology	15255	0376374
BJU International	Urology	6884	100886721
European Urology	Urology	4547	7512719
International Urogynecology Journal	Urology, Gynecology	2066	101567041
Circulation	Vascular Diseases, Cardiology	276579	0147763
Stroke	Vascular Diseases, Brain	76951	0235266
Hypertension	Vascular Diseases	18340	7906255
Journal of Vascular Surgery	Vascular Diseases, General Surgery	9848	8407742
Thrombosis Research	Vascular Diseases	4115	0326377
Journal of Thoracic and Cardiovascular Surgery	Vascular Diseases, Pulmonary Medicine, Cardiology, General Surgery	4072	0376343
Current Opinion in Nephrology and Hypertension	Vascular Diseases, Nephrology	1681	9303753
Journal of Midwifery and Women's Health	Women's Health, Obstetrics, Nursing	2482	100909407
