# Supplementary material for: PubMed's core clinical journals filter: redesigned for contemporary clinical impact and utility
Source: J Med Libr Assoc. 2023 Jul 10;111(3):665–76. doi: 10.5195/jmla.2023.1631 (PMC10361554; doi:10.5195/jmla.2023.1631)
Supplement: Supplementary file 7 — Appendix G: Fifteen Subjects with newly added Journals Ranked #1 [file jmla-111-3-665-s07.pdf]

Appendix G. Fifteen NLM Broad Subjects had newly added journals Ranked #1 in Clinical Usage

These 15 subjects with their newly added journals ranked #1 in Clinical Usage for that subject	
1. Communicable Diseases (*Clinical Infectious Diseases*)	9. Neurology (*Journal of Neurology, Neurosurgery & Psychiatry)*
2. Education (*Academic Medicine)*	10. Neurosurgery (*Journal of Neurology, Neurosurgery & Psychiatry)*
3. Emergency Medicine (*Journal of Emergency Medicine*)	11. Nutritional Sciences (*Breastfeeding Medicine*)
4. Gastroenterology (*American Journal of Gastroenterology*)	12. Ophthalmology (*JAMA Ophthalmology*)
5. Geriatrics (*Journal of the American Geriatrics Society*)	13. Otolaryngology *(JAMA Otolaryngology-Head & Neck Surgery)*
6. Hospitals (*Infection Control & Hospital Epidemiology*)	14. Pathology *(Modern Pathology)*
7. Neoplasms (*Journal of Clinical Oncology*)	15. Therapeutics (*Medical Letter on Drugs & Therapeutics*)
