# Supplementary material for: PubMed's core clinical journals filter: redesigned for contemporary clinical impact and utility
Source: J Med Libr Assoc. 2023 Jul 10;111(3):665–76. doi: 10.5195/jmla.2023.1631 (PMC10361554; doi:10.5195/jmla.2023.1631)
Supplement: Supplementary file 8 — Appendix H: Search strategy to retrieve the new CUJ [file jmla-111-3-665-s08.pdf]

Appendix H

Clinically Useful Journals PubMed Search Strategy

"AACN Adv Crit Care"[Journal] OR "Acad Emerg Med"[Journal] OR "Acad Med"[Journal] OR "Addict Behav"[Journal] OR "Age Ageing"[Journal] OR "AJR Am J Roentgenol"[Journal] OR "Allergy"[Journal] OR "Am Fam Physician"[Journal] OR "Am Heart J"[Journal] OR "Am J Epidemiol"[Journal] OR "Am J Gastroenterol"[Journal] OR "Am J Hematol"[Journal] OR "Am J Kidney Dis"[Journal] OR "Am J Med Genet A"[Journal] OR "Am J Med"[Journal] OR "Am J Nurs"[Journal] OR "Am J Obstet Gynecol"[Journal] OR "Am J Prev Med"[Journal] OR "Am J Psychiatry"[Journal] OR "Am J Respir Crit Care Med"[Journal] OR "Am J Sports Med"[Journal] OR "Am J Surg Pathol"[Journal] OR "Am J Med Sci"[Journal] OR "Anesth Analg"[Journal] OR "Ann Allergy Asthma Immunol"[Journal] OR "Ann Emerg Med"[Journal] OR "Ann Intern Med"[Journal] OR "Ann Neurol"[Journal] OR "Ann Oncol"[Journal] OR "Ann Pharmacother"[Journal] OR "Ann Surg"[Journal] OR "Ann Surg Oncol"[Journal] OR "Ann Rheum Dis"[Journal] OR "Ann Thorac Surg"[Journal] OR "Arch Dis Child"[Journal] OR "Arch Dis Child Fetal Neonatal Ed"[Journal] OR "Arch Phys Med Rehabil"[Journal] OR "Arthritis Rheumatol"[Journal] OR "Arthritis Care Res (Hoboken)"[Journal] OR "Arthroscopy"[Journal] OR "Autoimmun Rev"[Journal] OR "Best Pract Res Clin Rheumatol"[Journal] OR "Biol Psychiatry"[Journal] OR "BJOG"[Journal] OR "BJU Int"[Journal] OR "Blood"[Journal] OR "BMJ"[Journal] OR "Bone Marrow Transplant"[Journal] OR "Brain"[Journal] OR "Breastfeed Med"[Journal] OR "Br J Anaesth"[Journal] OR "Br J Cancer"[Journal] OR "Br J Dermatol"[Journal] OR "Br J Haematol"[Journal] OR "Br J Ophthalmol"[Journal] OR "Br Med Bull"[Journal] OR "CA Cancer J Clin"[Journal] OR "Cancer"[Journal] OR "Cancer Treat Rev"[Journal] OR "Catheter Cardiovasc Interv"[Journal] OR "Chest"[Journal] OR "Circulation"[Journal] OR "Clin Biochem"[Journal] OR "Clin Biomech (Bristol, Avon)"[Journal] OR "Clin Gastroenterol Hepatol"[Journal] OR "Clin Infect Dis"[Journal] OR "Clin Obstet Gynecol"[Journal] OR "Clin Pharmacol Ther"[Journal] OR "Clin Ther"[Journal] OR "Clin Podiatr Med Surg"[Journal] OR "CMAJ"[Journal] OR "Comput Inform Nurs"[Journal] OR "Crit Care Med"[Journal] OR "Curr Opin Cardiol"[Journal] OR "Curr Opin Gastroenterol"[Journal] OR "Curr Opin Nephrol Hypertens"[Journal] OR "Curr Opin Pediatr"[Journal] OR "Curr Opin Rheumatol"[Journal] OR "Diabetes Care"[Journal] OR "Diabetes Res Clin Pract"[Journal] OR "Diagn Microbiol Infect Dis"[Journal] OR "Dig Dis Sci"[Journal] OR "Dis Colon Rectum"[Journal] OR "Drug Alcohol Depend"[Journal] OR "Early Hum Dev"[Journal] OR "Epilepsia"[Journal] OR "Epilepsy Behav"[Journal] OR "Europace"[Journal] OR "Eur Heart J"[Journal] OR "Eur J Cancer"[Journal] OR "Eur J Cardiothorac Surg"[Journal] OR "Eur J Heart Fail"[Journal] OR "Eur J Intern Med"[Journal] OR "Eur J Nucl Med Mol Imaging"[Journal] OR "Eur J Radiol"[Journal] OR "Eur Urol"[Journal] OR "Fertil Steril"[Journal] OR "Gastroenterology"[Journal] OR "Gastrointest Endosc"[Journal] OR "Gut"[Journal] OR "Gynecol Oncol"[Journal] OR "Head Neck"[Journal] OR "Headache"[Journal] OR "Health Aff (Millwood)"[Journal] OR "Heart"[Journal] OR "Heart Rhythm"[Journal] OR "Hepatology"[Journal] OR "Hum Pathol"[Journal] OR "Hum Reprod"[Journal] OR "Hypertension"[Journal] OR "Infect Control Hosp Epidemiol"[Journal] OR "Int J Antimicrob Agents"[Journal] OR "Int J Cancer"[Journal] OR "Int J Cardiol"[Journal] OR "Int J Clin Pract"[Journal] OR "Int J Obes"[Journal] OR "Int J Radiat Oncol Biol Phys"[Journal] OR "Int Urogynecol J"[Journal] OR "JAMA"[Journal] OR "JAMA Dermatol"[Journal] OR "JAMA Intern Med"[Journal] OR "JAMA Neurol"[Journal] OR "JAMA Ophthalmol"[Journal] OR "JAMA Otolaryngol Head Neck Surg"[Journal] OR "JAMA Pediatr"[Journal] OR "JAMA Psychiatry"[Journal] OR "JAMA Surg"[Journal] OR "J Healthc Qual"[Journal] OR "J Acquir Immune Defic Syndr"[Journal] OR "J Adv Nurs"[Journal] OR "J Allergy Clin Immunol"[Journal] OR "J Altern Complement Med"[Journal] OR "J Bone Joint Surg Am"[Journal] OR "J Card Fail"[Journal] OR "J Clin Endocrinol Metab"[Journal] OR "J Clin Gastroenterol"[Journal] OR "J Clin Neurosci"[Journal] OR "J Clin Oncol"[Journal] OR "J Clin Pathol"[Journal] OR "J Clin Psychol"[Journal] OR "J Clin Psychopharmacol"[Journal] OR "J Emerg

Med"[Journal] OR "J Foot Ankle Surg"[Journal] OR "J Gen Intern Med"[Journal] OR "J Hand Surg Am"[Journal] OR "J Hepatol"[Journal] OR "J Hosp Infect"[Journal] OR "J Hosp Med"[Journal] OR "J Infect"[Journal] OR "J Infect Dis"[Journal] OR "J Intern Med"[Journal] OR "J Invest Dermatol"[Journal] OR "J Med Genet"[Journal] OR "J Midwifery Womens Health"[Journal] OR "J Neurol Neurosurg Psychiatry"[Journal] OR "J Nurs Adm"[Journal] OR "J Obstet Gynecol Neonatal Nurs"[Journal] OR "J Occup Environ Med"[Journal] OR "J Oral Maxillofac Surg"[Journal] OR "J Orthop Sports Phys Ther"[Journal] OR "J Orthop Trauma"[Journal] OR "J Pain Symptom Manage"[Journal] OR "J Palliat Med"[Journal] OR "J Pediatr Gastroenterol Nutr"[Journal] OR "J Pediatr Hematol Oncol"[Journal] OR "J Pediatr Orthop"[Journal] OR "J Pediatr Surg"[Journal] OR "J Perinatol"[Journal] OR "J Psychopharmacol"[Journal] OR "J Subst Abuse Treat"[Journal] OR "J Surg Oncol"[Journal] OR "J Am Coll Cardiol"[Journal] OR "J Am Geriatr Soc"[Journal] OR "J Am Med Dir Assoc"[Journal] OR "J Am Med Inform Assoc"[Journal] OR "J Natl Cancer Inst"[Journal] OR "J Thorac Cardiovasc Surg"[Journal] OR "J Thromb Haemost"[Journal] OR "J Trauma Acute Care Surg"[Journal] OR "J Urol"[Journal] OR "J Vasc Surg"[Journal] OR "JPEN J Parenter Enteral Nutr"[Journal] OR "Kidney Int"[Journal] OR "Lancet"[Journal] OR "Laryngoscope"[Journal] OR "Leukemia"[Journal] OR "Liver Transpl"[Journal] OR "Med Care"[Journal] OR "Med Clin North Am"[Journal] OR "Med Lett Drugs Ther"[Journal] OR "Medicine (Baltimore)"[Journal] OR "Mod Pathol"[Journal] OR "Mol Genet Metab"[Journal] OR "Mov Disord"[Journal] OR "Muscle Nerve"[Journal] OR "Nephrol Dial Transplant"[Journal] OR "Neurology"[Journal] OR "Neurosurgery"[Journal] OR "N Engl J Med"[Journal] OR "Nursing"[Journal] OR "Obesity (Silver Spring)"[Journal] OR "Obstet Gynecol Surv"[Journal] OR "Obstet Gynecol"[Journal] OR "Oral Surg Oral Med Oral Pathol Oral Radiol"[Journal] OR "Otolaryngol Head Neck Surg"[Journal] OR "Pain"[Journal] OR "Pain Med"[Journal] OR "Patient Educ Couns"[Journal] OR "Pediatr Dermatol"[Journal] OR "Pediatr Infect Dis J"[Journal] OR "Pediatrics"[Journal] OR "Pharmacoepidemiol Drug Saf"[Journal] OR "Plast Reconstr Surg"[Journal] OR "Postgrad Med J"[Journal] OR "Prev Med"[Journal] OR "Prim Care"[Journal] OR "Psychiatr Serv"[Journal] OR "QJM"[Journal] OR "Radiographics"[Journal] OR "Radiology"[Journal] OR "Radiother Oncol"[Journal] OR "Respir Med"[Journal] OR "Semin Dial"[Journal] OR "Semin Nucl Med"[Journal] OR "Semin Perinatol"[Journal] OR "Semin Respir Crit Care Med"[Journal] OR "Semin Ultrasound CT MR"[Journal] OR "Sex Transm Dis"[Journal] OR "Sex Transm Infect"[Journal] OR "Soc Sci Med"[Journal] OR "South Med J"[Journal] OR "Spine (Phila Pa 1976)"[Journal] OR "Stat Methods Med Res"[Journal] OR "Stat Med"[Journal] OR "Stroke"[Journal] OR "Thorax"[Journal] OR "Thromb Res"[Journal] OR "Thyroid"[Journal] OR "Ultrasound Obstet Gynecol"[Journal] OR "Vaccine"[Journal] OR "World Neurosurg"[Journal]
